# Viral and Immunological Analytes are Poor Predictors of the Clinical Treatment Response in Kaposi’s Sarcoma Patients

**DOI:** 10.3390/cancers12061594

**Published:** 2020-06-16

**Authors:** Salum J. Lidenge, For Yue Tso, Yasaman Mortazavi, John R. Ngowi, Danielle M. Shea, Julius Mwaiselage, Charles Wood, John T. West

**Affiliations:** 1 Nebraska Center for Virology, Lincoln, NE 68583, USA; sjlidenge@yahoo.co.uk (S.J.L.); ftso2@unl.edu (F.Y.T.); yass.mortazavi@yahoo.com (Y.M.); danielle.shea@unl.edu (D.M.S.); 2 School of Biological Sciences, University of Nebraska-Lincoln, Lincoln, NE 68588, USA; 3 Department of Academics and Research, Ocean Road Cancer Institute, P. O. Box 3592 Dar es Salaam, Tanzania; jrngowi146@gmail.com (J.R.N.); jmwaiselage@yahoo.com (J.M.); 4 Department of Clinical Oncology, Muhimbili University of Health and Allied Sciences, P. O. Box 65001 Dar es Salaam, Tanzania; 5 Department of Biochemistry, University of Nebraska-Lincoln, Lincoln, NE 68588, USA

**Keywords:** Kaposi’s sarcoma, immune responses, neutralizing antibody, cytokine, T-cells, treatment response, HIV-1, KSHV, sub-Saharan Africa

## Abstract

Kaposi’s sarcoma-associated herpes virus (KSHV) is the etiologic agent for Kaposi’s sarcoma (KS). The prognostic utility of KSHV and HIV-1 (human immunodeficiency virus) viremia as well as immunological parameters in clinical management of participants with KS is unclear. The objective of this study was to investigate viral and immunological parameters as predictors of KS treatment responses in participants with KS from sub-Saharan Africa (SSA). Plasma KSHV-DNA, HIV-1 viral load, total anti-KSHV antibody, KSHV-neutralizing antibody (nAb), cytokine/chemokine levels, and T-cell differentiation subsets were quantified before and after KS treatment in 13 participants with KS and in 13 KSHV-infected asymptomatic control individuals. One-way analysis of variance and the Mann-Whitney t-test were used to assess differences between groups where *p*-values < 0.05 were considered significant. Subjects with patch and plaque KS lesions responded more favorably to treatment than those with nodular lesions. Pre-treatment and post-treatment levels of plasma KSHV-DNA, HIV-1 viral load, KSHV-Ab responses, cytokines, and T-cell populations did not predict the KS treatment response. Elevated KSHV-humoral and cytokine responses persisted in participants with KS despite a clinical KS response. While patch and plaque KS lesions were more common among treatment responders, none of the analyzed viral and immunological parameters distinguished responders from non-responders at baseline or after treatment.

## 1. Introduction

Kaposi’s sarcoma (KS), which is a multifocal angio-proliferative sarcoma commonly found on skin and mucosal surfaces, is caused by the Kaposi’s sarcoma-associated herpes virus (KSHV) or human herpes virus-8 (HHV-8) [1]. KSHV infection is endemic in sub-Saharan Africa (SSA) with prevalence as high as 90% in some countries [2,3,4]. Before the AIDS epidemic, HIV negative African-endemic KS (EnKS) comprised 4–10% of African adult cancers [5,6]. With the ongoing HIV/AIDS epidemic, AIDS-associated/epidemic KS (EpKS) has become the most common cancer in HIV-1 infected African individuals [7,8].

In developed countries, anti-retroviral therapy (ART) has led to significant decline in KS incidence in HIV-1 infected individuals, even though recent reports demonstrate that KS still occurs in individuals with reconstituted CD4^+^ T-cell counts and a suppressed HIV-1 plasma viral load [9,10,11,12,13]. A similar decline in KS incidence has not been observed in SSA where KS remains one of the top five cancers despite of increased ART coverage, uptake, and adherence [9,14,15]. Lack of early disease recognition, advanced presentation, and lack of effective treatments all contribute to high KS morbidity and mortality [16,17,18].

KS treatment in SSA consists of conventional chemotherapy and/or radiotherapy [19,20,21,22]. The readily available KS treatments are suboptimal with no curative effects [17,21,23]. First-line KS treatments employed in developed countries, such as liposomal doxorubicin, alone or in combination with other drugs, are not readily accessible [24]. With currently available KS treatments, approximately 50% of African participants with KS achieve some degree of remission [17,21]. Unfortunately, approximately 50% of initially remitting patients experience recurrence within 6 months post-treatment [21]. Factors that correlate with KS treatment responses are not currently understood. Since KS is associated with immune suppression, as shown by the high incidence of KS in HIV-1 co-infected individuals, it is possible that immunological differentials before and after KS treatment would associate with response to treatment. Identification of pre-treatment factors associated with a response might provide prognostic insight whereas differentials in markers post-treatment might suggest mechanisms associated with KS control. Therefore, investigation of participants with KS before and after treatment could be crucial for developing more effective management strategies for KS.

Our recent analysis of immunological responses in participants with EpKS and EnKS prior to initiation of treatment revealed differentials and potential dysregulation in antibody and cytokine responses in both participants with EpKS and EnKS [25]. Cytokines associated with increased antibody production (IL-6, IL-5, and IL-10) as opposed to inflammation and cellular responses [26,27,28,29] were significantly elevated in participants with KS at baseline. Since KS treatment with chemotherapy or radiotherapy can result in remission, we hypothesized that changes in viral and immunological parameters before and after treatment would differentiate responders from non-responders. Such changes could be applied to better stratify patients and improve KS management. However, our study reveals immune dysregulation persists in treated participants with KS independently of the therapeutic outcome.

## 2. Results

### 2.1. Characteristics of the Study Subjects

To investigate viral and immunological profiles as predictors of the KS treatment response, a total of 26 individuals (eight KS responders and five non-responders with complete clinical information and 13 KSHV-infected asymptomatic controls, six HIV-1^−^ and seven HIV-1^+^) were recruited. The two control groups were comparable in age, gender, CD4 count, and KSHV status (Table 1). Similarly, there was no statistically significant difference between responders and non-responders in age, gender, KS lesion morphotype, duration of KS, or ART use and CD4 count at baseline. While the comparison of CD4 count did not reveal significant differences between groups of controls and participants with KS, KS non-responders had significantly lower CD4 count when compared to normal controls (HIV-1^−^) (*p* = 0.03). Participants with KS and controls had comparable duration to ART. Plasma HIV-1 viremia was also comparable between HIV-1^+^ controls and participants with KS despite detectable HIV-1 in a few of the participants with KS (Table 2). The major KS treatment modality was Adriamycin/Bleomycin/Vinblastine (ABV) chemotherapy except for two cases where radiotherapy alone was applied to patch and plaque KS lesions on the extremities (Table 1).

### 2.2. Similar Viral Profiles in Responders and Non-Responders of KS Treatment

Although KSHV is mostly a cell associated virus, our previous analysis of KSHV detection in a peripheral blood mononuclear cell (PBMC) revealed a variable detection pattern and lacked association with anti-KSHV antibody responses [25]. Due to high anti-KSHV antibody responses in KS patients, we investigated KSHV viremia and antibody responses as predictors of the KS treatment response. DNase-I treated plasma was used to quantify KSHV virion DNA before and after treatment. Only three treatment responders and three non-responders had detectable KSHV viremia at baseline, and, when detectable, the baseline quantity of KSHV virions in plasma was comparable between responders and non-responders. However, after KS treatment, only one of six individuals (17%) maintained detectable KSHV plasma viremia. The rest became undetectable (Table 2). KSHV viremia was below the detection limit for most of the plasma samples. Importantly, KSHV viremia after treatment did not correlate with the therapeutic response.

For the participants with EpKS and HIV-1^+^ asymptomatic controls, the HIV-1 plasma viral load was largely undetectable before and after KS treatment, and CD4^+^ T-cell counts in the HIV-1^+^ subjects were similar to those in HIV-1^−^ controls (Table 1). Individuals with detectable plasma HIV-1 were either on ART for less than a month (2-individuals) or were experiencing ART failure (1-individual). With continued ART use, all patients attained HIV-1 viral suppression except one (21161) who was switched to the second line ART (Table 2). However, there were no statistically significant differences in pre-treatment or post-treatment CD4 count or HIV-1 plasma viral load between responders and non-responders or between controls and participants with KS. Importantly, none of the variables in Table 1 and Table 2 are significantly associated with the KS treatment response by logistic regression analysis.

### 2.3. Humoral Response Differentials between KS Treatment Responders and Non-Responders

**Figure 1:**
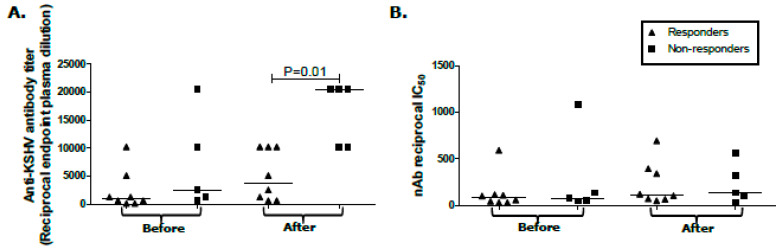
KSHV-specific humoral responses before and after treatment. (**A**) Immunofluorescence assay for total anti-KSHV antibody titers in plasma of participants with KS showing responders and non-responders before and after KS treatment (reciprocal endpoint plasma dilution). (**B**) KSHV-neutralizing antibody (nAb) titer in plasma of participants with KS showing responders and non-responders before and after KS treatment, presented as a reciprocal of 50% inhibitory concentration (IC_50_). Plasma samples that were nAb-positive at 1:50 dilution were re-assayed in two-fold dilutions of plasma from 1:50 to 1:800 to define the IC_50_. KSHV-seropositive samples with less than 50% KSHV neutralization at 1:50 dilution were assigned a value of 30 in reciprocal IC_50_ plots.

We previously reported high levels of total anti-KSHV Ab and KSHV-nAb in participants with KS prior to cancer therapy [25]. In this case, we tested whether titers of these Ab responses at baseline, or their changes in response to therapy, correlated with the outcome. Neither high nor low Ab titer at baseline was associated with the treatment response. However, non-responders had higher anti-KSHV Ab levels after treatment, which may be indicative of continuing low levels of viral expression without resulting in detectable plasma viremia (*p* = 0.01, Figure 1A). High anti-KSHV Ab titers have also been shown to correlate with KS disease [25,30]. The median KSHV-nAb titers and high or low KSHV-nAb titers before or after treatment also did not correlate with the response outcome (Figure 1B). Overall, despite undetectable KSHV plasma viremia, KSHV-specific humoral responses are still high after treatment but failed to differentiate responders from non-responders.

### 2.4. Cytokine/Chemokine Levels in Responders and Non-Responders Unaffected by KS Treatment

**Figure 2:**
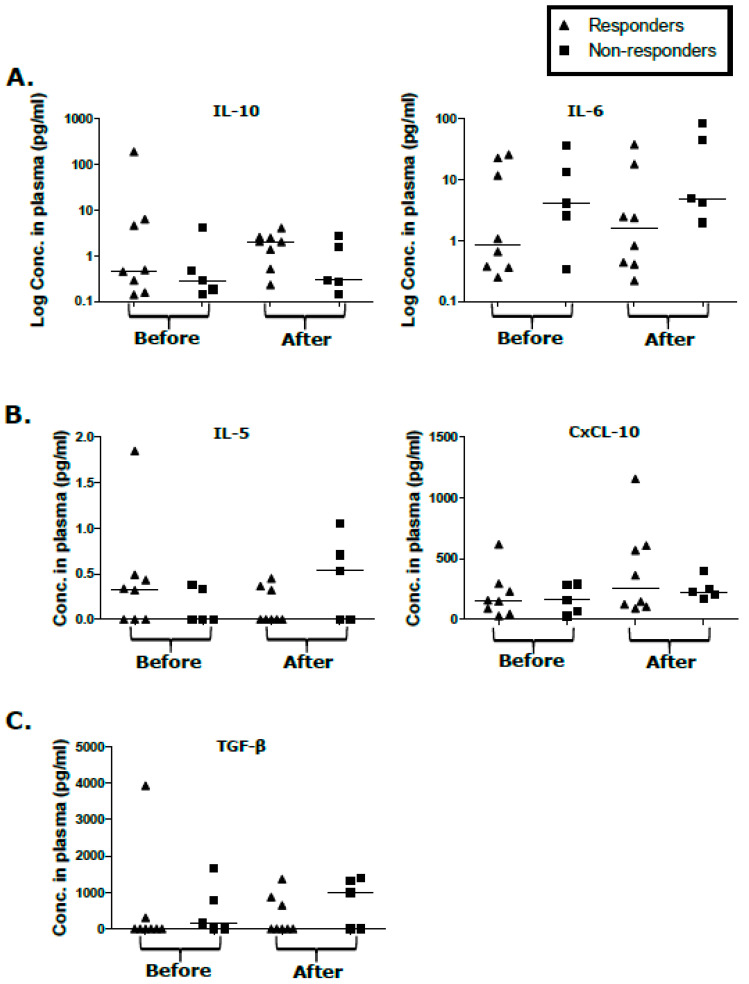
Cytokine/chemokine responses in plasma of participants with Kaposi’s sarcoma (KS) showing responders and non-responders before and after KS treatment. (**A**) Interleukin-10 (IL-10), interleukin-6 (IL-6), (**B**) interleukin-5 (IL-5), chemokine CXCL10, and (**C**) transforming growth factor-β (TGF-β).

We recently reported IL-5, IL-10, IL-6, CxCL-10, and TGF-β elevation in both participants with EpKS and EnKS when compared to KSHV-infected asymptomatic controls prior to cancer therapy [25]. In this case, we tested whether baseline cytokine levels (high or low), or temporal changes in cytokine levels over KS treatment, predicted the KS treatment response. The average levels, the high or low levels of regulatory/inhibitory cytokines, IL-10 and TGF-β, and the anti-inflammatory cytokine, IL-5, did not vary significantly between responders and non-responders (Figure 2A–C). Similarly, the average levels of IL-6 and CxCL-10 did not correlate with the treatment response (Figure 2A,B). High versus low partitions of IL-6 and CxCL-10, before or after treatment, also did not correlate with the treatment response (Figure 2A,B). Overall, the elevation of inhibitory and regulatory cytokines in participants with KS after treatment compared to non-disease controls implicates persistent immune dysregulation.

### 2.5. T-Cell Populations Are Not Differential between KS Treatment Responders and Non-Responders

**Figure 3:**
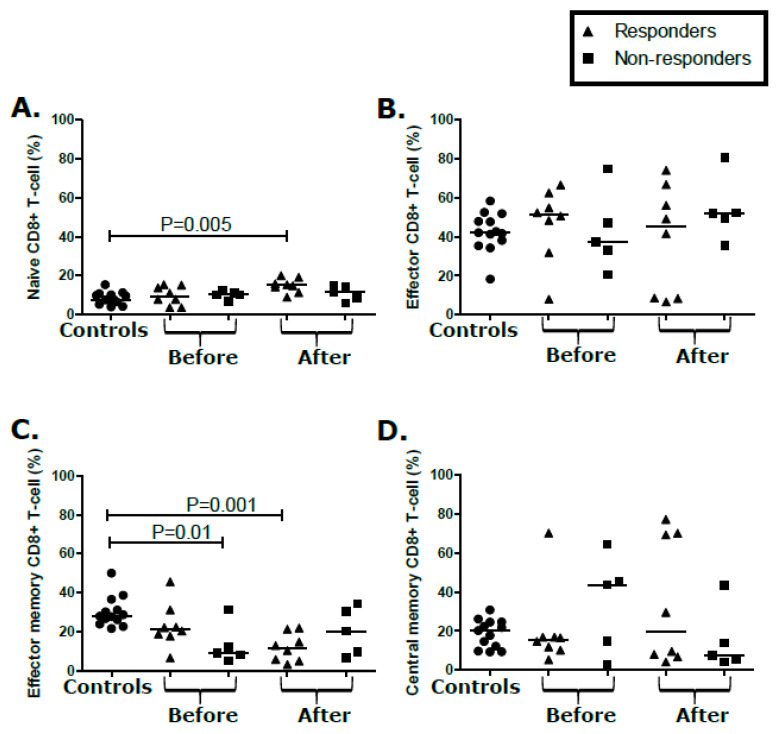
T-cell population (CD8^+^) analysis from peripheral blood mononuclear cells (PBMCs). Percentage of CD8^+^ T-cell population expressing markers of (**A**) naïve, (**B**) effector, (**C**) effector memory, and (**D**) central memory CD8^+^ T-cells in asymptomatic controls and responder and non-responders before and after treatment.

As a result of antigenic stimulation, T-cells undergo phenotypic changes such as activation, differentiation, and proliferation [31,32]. We immuno-phenotyped peripheral blood T-cell populations from participants with KS before and after treatment to investigate whether T-cell subsets were associated with the KS treatment response. Naïve [CD197^+^/CD45RO^−^ (T_N_)], effector [CD197^−^/CD45RO^−^ (T_E_)], effector memory [CD197^−^/CD45RO^+^ (T_EM_)], central memory [CD197^+^/hCD45RO^+^ (T_CM_)] CD4^+^, and CD8^+^ T-cell populations were quantified by flow cytometry. Their activation (CD38^+^/Human Leucocyte Antigen/HLA-DR^+^), senescence (CD57^+^/CD28^−^/hCD27^−^), and proliferation (Ki67^+^) profiles were also investigated. Compared to controls, there was an increase in proportions of T_N_ (*p* = 0.005) and a decrease of T_EM_ CD8^+^ T-cells (*p* = 0.001) in KS responders (Figure 3A,C, respectively). In participants with KS, T_E_ and T_CM_ CD8^+^ T-cells were comparable to controls (Figure 3B,D). No changes were noted in proportions of CD8^+^ T-cell phenotypes over the course of treatment (Figure 3A–D). Similarly, none of the proportions of CD8^+^ T_N_, T_E_, T_EM_, and T_CM_ at baseline, or after treatment, correlated with the KS treatment response (Figure 3A–D). Importantly, having high or low proportions CD8^+^ T_N_, T_E_, T_EM_, and T_CM_ before and after treatment also showed no significant correlation with the treatment response (Figure 3A–D).

**Figure 4:**
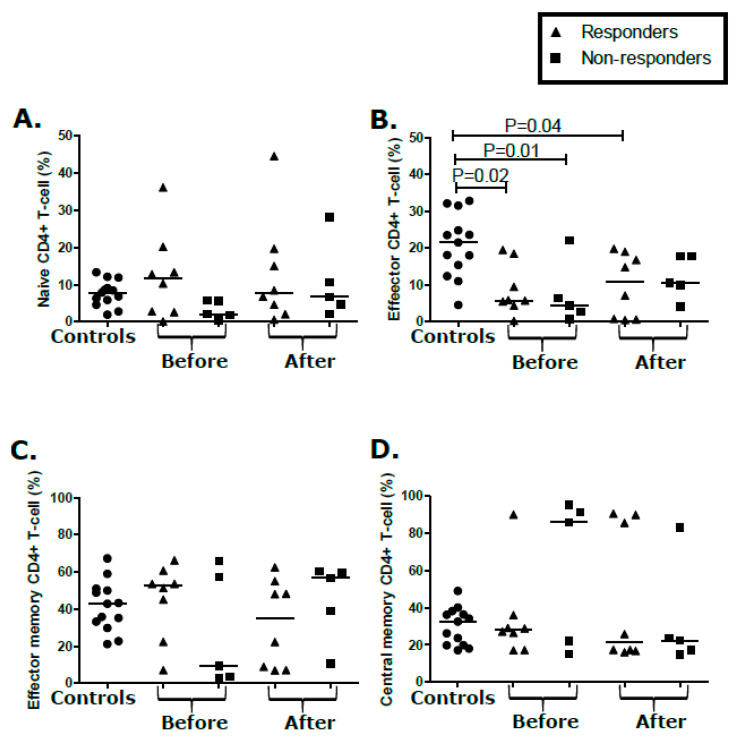
T-cell population (CD4^+^) analysis from peripheral blood mononuclear cells (PBMCs). Percentage of CD4^+^ T-cell population expressing markers of (**A**) naïve, (**B**) effector, (**C**) effector memory, and (**D**) central memory CD4^+^ T-cells in asymptomatic controls and responder and non-responders before and after treatment.

**Figure 5:**
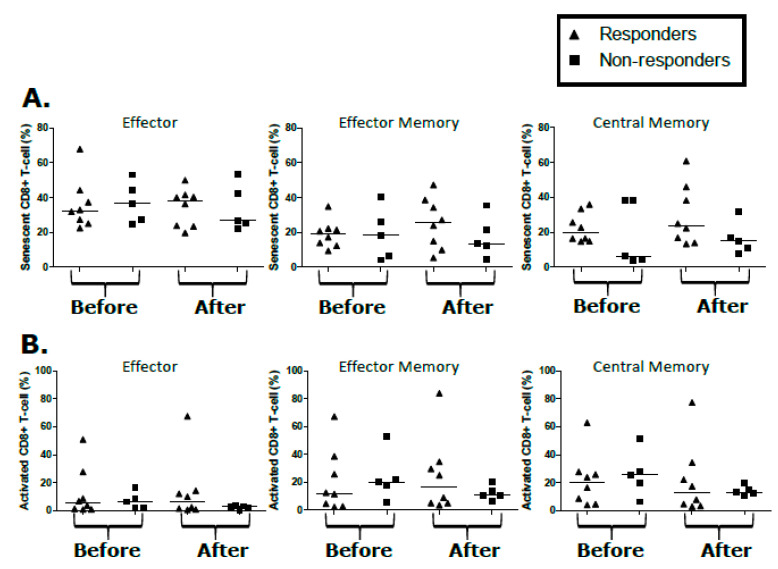
T-cell population (CD8^+^) analysis from peripheral blood mononuclear cells (PBMCs). (**A**) Percentage of CD8^+^ T-cell expressing senescence markers (CD57^+^/CD28^−^/CD27^−^) among subsets of CD8^+^ T-cells in effector, effector memory, and central memory CD8^+^ T-cells in responders and non-responders before and after KS treatment. (**B**) Percentage of CD8^+^ T-cell expressing activation markers (CD38^+^ and HLA-DR^+^) among subsets of CD8^+^ T-cells in effector, effector memory, and central memory CD8^+^ T-cells in responders and non-responders before and after KS treatment.

In the CD4^+^ compartment, proportions of T_N_, T_EM_, and T_CM_ in participants with KS were comparable to controls (Figure 4A,C,D). However, there were decreased CD4^+^ T_E_ (*p* = 0.01) in KS responders when compared to the controls (Figure 4B), but this differential was maintained through treatment in comparison of the baseline to follow-up (*p* = 0.04, Figure 4B). There were no temporal/treatment-associated changes in the CD4^+^ T-cell phenotypes over the course of treatment (Figure 4A–D). Similarly, none of the proportions of CD4^+^ T_N_, T_E_, T_EM_, and T_CM_ at baseline or after treatment correlated with the KS treatment response (Figure 4A–D). No significant changes were detected in activation, proliferation, and senescence markers in either the CD8^+^ or CD4^+^ T-cell populations of responders and non-responders from pre-treatment or post-treatment time points (Figure 5 and Supplementary Figures S1 and S2). Overall, despite a demonstrated KS clinical treatment response, the proportions T-cell subsets before and after treatment were not correlative or predictive of the KS treatment response.

## 3. Discussion

To our knowledge, this is the first study to investigate viral and immunological parameters as predictors of treatment responses in participants with KS receiving local standard-of-care treatment in SSA. KS presentation is intrinsically variable due to dissimilarities in KS symptomology coupled to variances in HIV-1 immune and virological parameters. We sought to investigate outcome associations in a typically variable KS cohort to identify potential parameters associated with treatment response. We have quantified KSHV and HIV-1 viremia, KSHV-humoral responses, cytokine expression, and T-cell immunophenotypes before and after treatment to identify factors that correlate with the KS clinical response, or lack thereof. An advanced cancer stage is known to correlate with poor treatment outcome [19,20,21,22]. In contrast, early diagnosis and treatment associates with improved clinical response and reduced morbidity [16,17,18]. Our analysis of factors associated with treatment response also revealed a trend toward a favorable treatment outcome for individuals presenting with patch and plaque KS lesions at baseline, and the nodular lesion morphotypes were associated with KS non-responders [33]. This highlights the importance of early identification, diagnosis, and treatment of KS to achieve better treatment outcomes in SSA.

Although KSHV viremia has been investigated as a tumor biomarker [34,35], a study of 684 HIV-1^+^ individuals with KS and Multicentric Castleman’s Disease (MCD) showed plasma KSHV viral load did not predict survival in T0 or T1-staged participants with KS [36,37]. Consistent with these findings, KSHV viremia before or after treatment did not correlate with the KS treatment response in our study. KSHV viremia also did not associate with HIV-1 co-infection [25]. However, plasma KSHV viremia decreased as a consequence of chemotherapy/radiotherapy, which is consistent with the report by Lin et al. [36]. Additionally, CD4^+^ T-cell count and HIV-1 viremia did not predict the KS treatment response. Similarly, a Zambian study on participants with early KS, to evaluate the longitudinal response to KS treatment with ART alone, found that HIV-1 viremia and CD4 count before and after treatment also did not differentiate KS responders from non-responders (Ngalamika et al., in submission). The lack of outcome association with both CD4^+^ T-cell count and HIV-1 viremia is also supported by recent reports of incident KS in individuals with high CD4^+^ T-cell counts and suppressed HIV-1 viral load [9,10,11,12,13]. Since our transcriptomic analysis of EpKS lesions revealed no evidence for HIV-1 transcripts directly in KS lesions (Lidenge et al. in submission), the lack of outcome association with both the CD4^+^ T-cell count and HIV-1 viremia could be due to an indirect impact of HIV on immune function as opposed to simply CD4^+^ T-cell depletion.

Changes in immune parameters such as CD4^+^, CD8^+^ T-cells, NK, and myeloid derived suppressor cell populations have been suggested to be biomarkers for lung cancer and non-Hodgkin’s lymphoma treatment responses [38,39]. CD4^+^ T-cell help and cytotoxic activity of CD4^+^ and CD8^+^ T-cells play an important role in eliminating infected cells and cancer cells [40]. Poor KS treatment outcomes could result from reductions in CD4^+^ T_E_ and CD8^+^ T_EM_ cells in participants with KS. However, reduction in CD4^+^ T_E_ and CD8^+^ T_EM_ was also evident in responders suggesting possible T-cell qualitative differentials between responders and non-responders. Unfortunately, whether decreases in CD4^+^ T_E_ and CD8^+^ T_EM_, or the noted increase in CD8^+^ naïve T-cells corresponded to decreased KSHV Ag-specific effector functions could not be determined. Assessment of differentials in functionality of T-cells between treatment responders and non-responders will be important in identifying predictors of the KS treatment response.

Cytokine dysregulation has often been associated with KS development [41,42]. However, longitudinal studies of cytokine responses in African participants with KS are lacking. Only a few studies investigated the role of cytokines in predicting the KS treatment response in US-based patients [43,44]. In one study, seven patients on ART were treated with rapamycin. Analysis of viral and immunological parameters revealed no significant changes in plasma KSHV and HIV-1 viral load, CD4 count, IL-6, and VEGF levels between KS responders and non-responders [43]. Similarly, a phase II trial of imatinib in participants with EpKS did not find significant association between the cytokines (IL-6, Rantes, IFN-γ, and basic fibroblast growth factor) analyzed and the KS treatment response [44]. Despite differences in KS treatment regimens between US and SSA, we consistently found that the IL-6, plasma KSHV, HIV-1 viral load, and CD4 count are not associated with the KS treatment response. Importantly, our study has not found significant association of outcome with any variance in IL-10, IL-5, and CxCL10 that were not previously investigated. However, granulocyte colony stimulating factor (G-CSF) and hepatocyte growth factor (HGF) were reported to correlate with the KS treatment response by Tedeschi et al. in a cohort of Italian participants with advanced EpKS [37]. Unfortunately, incompatibility in assay platforms (Millipore versus BD-CBA) and limited blood samples from participants with advanced KS prevented us from investigating G-CSF and HGF in this study. Overall, the Th2 skewing of cytokines we previously reported in pre-treated African participants with KS did not change significantly despite the demonstrated clinical KS response, and will not be good markers for the treatment response [25].

In acute viral infections, nAb responses often correlate with protection or control. However, for Epstein-Barr virus (EBV) and KSHV, high antibody titers are often associated with disease [45,46]. Our analysis of pre-treatment KSHV-nAb revealed higher prevalence and titers of nAb in both participants with EpKS and EnKS than in asymptomatic controls [25,30]. In this case, despite decreased KSHV viremia, total anti-KSHV and KSHV-nAb in participants with KS remained persistently high in both responders and non-responders. This is similar to the results observed from the analysis of participants with early KS treated with ART only (Ngalamika et al. in submission). It is possible that the high KSHV-humoral responses result from continued production of antibody-associated cytokines IL-6, IL-5, and IL-10 evident in both studies, even though the Ab response does not appear to contribute to KS control.

It is puzzling that none of the immune parameters assessed predicted KS treatment response despite the strong association between KS and immune suppression. Furthermore, it was noted that a reduction in size and number of KS lesions after treatment did not correlate with the magnitude of the immunological responses measured to date. This implies that the response requires modulation of the qualitative or functional aspects of immune control. The lack of significant changes potentially explains the short-lived clinical responses and high recurrence rates reported in other studies [13,21]. As suggested by Roshan et al., weak, and individualized T-cell functional responses (IFN-γ) against KSHV, coupled with a lack of immunodominant epitopes, seem to be common features in participants with KS and in asymptomatic controls [47]. It is possible that differences between treatment responders and non-responders lie in the differences in the T-cell functional repertoire against KSHV and KS. Other host factors such as the HLA type or efficiency of viral/tumor Ag presentation may also play a role in determining the response to treatment in participants with KS. Further functional studies over the course of treatment will be essential to define relationships between host factors and functional T-cell responses that determine the KS treatment outcome. The extent of KS disease prior to initiation of treatment appears to be more important than viral and immunological parameters assessed later in the disease.

A limitation of our study is the small sample size. The stringent recruitment criteria to ensure that only confirmed KS cases are analyzed has limited our sample size and, hence, the depth of our analyses. Thus, it is unlikely that subtle immunological differences between the treatment responders and non-responders can be detected. Additionally, late disease presentation due to the lack of mechanisms for identification of early KS, together with losses during follow-up, have limited our sample size and the depth of our investigations. Larger studies with close patient follow-up are warranted to fully characterize viral and immunological factors associated with the KS treatment response. However, this study highlights the fact that immune dysregulation in participants with KS persists despite a reduction in size and number of lesions in some participants. Additionally, the tested viral and immunological parameters did not predict the KS treatment response in SSA.

## 4. Materials and Methods

### 4.1. Study Design, Subjects, and Samples

The cohort was comprised of 26 study subjects who were ≥18 years of age and of both genders from Ocean Road Cancer Institute (ORCI), Dar es Salaam including 13 participants with KS and 13 KSHV-infected asymptomatic controls. Seven of the controls were HIV-1^+^ and six were HIV-1^−^. Consenting subjects with complete clinical and treatment information were recruited. Only participants with KS who were newly diagnosed, histologically confirmed, and KS biopsy KSHV-DNA PCR positive at baseline were included. This is to avoid the approximately 35% misdiagnosed cases resulting from only clinical and/or histopathology diagnosed cases in SSA [48,49]. Peripheral blood samples were collected at baseline and at 3–12 months post-therapy cessation. Plasma was HIV-1 tested and the KSHV viral load was quantified by real-time PCR. KS tumor biopsy samples (4 mm) were collected (baseline only). All study procedures were approved by the institutional review boards from the Tanzania National Institute for Medical Research, Ocean Road Cancer Institute, and the University of Nebraska-Lincoln (UNL), IRB number: 20141014709FB.

### 4.2. Kaposi’s Sarcoma Response Criteria

According to ORCI treatment guidelines, all participants with EpKS, who were not on ART, began ART immediately after diagnosis. Depending on the extent of KS disease, patients are then treated with combined chemotherapy consisting of six cycles of adriamycin, bleomycin, and vinblastine/vincristine (ABV) or radiotherapy (XRT). In this study, the KS treatment response was determined 3 months after completion of ABV chemotherapy or XRT. Some KS treatment responders maintained their remission status up to 12 months after completion of treatment. A responder was defined under the AIDS Clinical Trial Group (ACTG) clinical response criteria for a complete and partial response while non-responders included stable and progressive disease [50]. For statistical analysis, all patients were defined as either responders or non-responders. In cases where patch, plaque, and nodular KS lesions existed in the same patient, the most advanced KS lesion was used to define the lesion morphotype.

### 4.3. HIV-1 Status and HIV-1 and KSHV Quantification in Plasma and Tumor Tissues

HIV-1 infection status and quantification of plasma HIV-1 viral load before and after treatment were performed as previously described [25]. Extraction of KSHV viral DNA from plasma, and KS tumor biopsies followed by real-time PCR amplification of KSHV-ORF26 were conducted as previously described [25].

### 4.4. rKSHV.219 Production, KSHV Serology, and Neutralization Assays

The rKSHV.219 was generated and tittered on HEK293T cells as previously described [25,51]. Levels and titers of the KSHV antibody (Ab) and neutralizing antibody (nAb) were determined as previously described [25]. Heat inactivated plasma was incubated at 1:50 dilution with rKSHV.219 at 37 °C for one hour. A virus-plasma mixture was used to infect HEK293T cells at 37 °C for 72 h. Flow cytometry was used to quantify infection. Plasma samples demonstrating >50% inhibition of infection compared to a negative control plasma were categorized as neutralizing. All samples that were KSHV-nAb positive at the 1:50 dilution were re-assayed in two-fold dilutions of plasma from 1:50 to 1:800 to define the 50% inhibitory concentration (IC_50_).

### 4.5. Multiplex Bead-Based Immunoassay

Plasma cytokines and chemokines were quantified with Becton-Dickinson Cytometric-Bead-Array (CBA) FlexSet kits, according to the manufacturer’s protocol. The standard sensitivity array (picograms/mL) included interleukin-4 (IL-4), chemokine CxCL10, interleukin-5 (IL-5), and transforming growth factor-β (TGF-β). The enhanced sensitivity array (femtograms/mL) included interferon-ɣ (IFN-ɣ), interleukin-1β (IL-1β), interleukin-6 (IL-6), interleukin-12p70 (IL-12p70), interleukin-17A (IL-17A), interleukin-10 (IL-10), and tumor necrosis factor- α (TNF-α). Raw data were collected on an Accuri C6 Plus cytometer (BD-Biosciences, San Jose, CA, USA) and analyzed with FlowJo version-10 (TreeStar, Ashland, OR, USA).

### 4.6. Flow Cytometry

To immunophenotype cell populations, PBMC were thawed, washed, split into two panels, and stained for 10 min in the dark at room temperature with antibodies against the following human surface markers: CD3 Percp.Cy5.5 (BD-Biosciences, San Jose, CA, USA), CD4, CD8-FITC, CD45RO-APC, CD197-AlexaFluor-700, CD38-BV510, HLA-DR-PE.Cy7, CD28-BV711, CD27 PE-Dazzle-594, CD57-APC, and CD107a BV421(Biolegend, San Diego, CA, USA). The cells were fixed using Perm/fix (BD-Biosciences, San Jose, CA, USA) for 20 min at 4 °C in the dark, and then stained for intracellular Ki67 (Biolegend, San Diego, CA, USA) or IFN-γ (Biolegend, San Diego, CA, USA). Raw data were quantified by FACSAria (BD-Biosciences, San Jose, CA, USA) and analyzed with FlowJo version-10.

### 4.7. Statistical Analysis

Viral nucleic acid detection in plasma, antibody, and cytokine responses as well as T-cell immunophenotypes were stratified into high and low levels before and after KS treatment. Correlation with the treatment response was analyzed. One-way analysis of variance with Tukey’s multiple comparison test and non-parametric Mann-Whitney t-test were employed to determine differences between groups. One control group was (KSHV^+^/HIV-1^+^) similar to EpKS patients in order to control for viral co-infection and investigate the effect of cancer (KS) in the background of HIV-1 co-infection on T-cell phenotypes while the other group of controls was (KSHV^+^, HIV-1^−^) similar to EnKS patients in order to control for KSHV infection and investigate the effect of cancer (KS) in the absence of HIV-1 co-infection on T-cell phenotypes. Similar analyses on cytokine and antibody responses have been reported previously [25]. The effect of the variables as predictors of KS treatment response was determined by logistic regression using SAS version 9.4 software (SAS Institute, Cary, NC, USA). GraphPad Prism v5 (GraphPad Software, San Diego, CA, USA) was used for statistical analyses. All tests were 2-tailed, and *p*-values < 0.05 were considered significant.

## 5. Conclusions

In summary, there was a trend toward favorable treatment outcomes in patch and plaque stages of KS. Our study tested KSHV and HIV-1 viremia, CD4 T-cell counts, KSHV-humoral responses, various cytokine responses, and the magnitude of T-cell population subsets as predictors of the KS treatment response but did not find significant associations. An important finding of this study is that, despite favorable clinical responses in some participants with KS, immunological dysregulation observed at baseline, persisted in these patients in remission, and this likely contributes to the high KS recurrence rates.

## Figures and Tables

**Table 1:** Characteristics of the study cohort at baseline.

Variables	Kaposi’s Sarcoma-Associated Herpes Virus (KSHV) Infected Asymptomatic Controls		Participants with Kaposi’s Sarcoma (KS)		*p*-Values *
	HIV-1^−^ (*n* = 6)	HIV-1^+^ (*n* = 7)	KS Responders (*n* = 8)	KS Non-Responders (*n* = 5)	
Median age-years (Range)	49 (33–55)	36 (34–48)	38 (26–57)	44 (39–58)	0.05
Male gender (%)	4 (67)	3 (43)	4 (50)	5 (100)	0.07
Nodular KS lesions (%)	NA	NA	3 (37.5)	4 (80)	0.15
KS duration ** (Range)	NA	NA	12 (1–96)	6 (3–36)	0.68
Median CD4 count (Range)	620 (303–1398)	316 (124–675)	377.5 (130–887)	150 (91–150) ^	0.09
Plasma HIV-1 load (copies/mL)	NA	BDL	3.5E4 (BDL-1.5E5)	1.0E4 (BDL-1.5E4)	0.80
Anti-retroviral therapy (ART) duration *** (Range)	NA	12 (1–36)	8 (1–36)	24 (0.25–72)	0.52

* Comparison between KS responders and non-responders. ** Self-reported KS duration in months. *** ART duration in months. Applies for six KS responders only. Two KS responders were HIV negative KS patients. ^ Comparison between KSHV infected asymptomatic controls (HIV-1^−^) and KS non-responders; *p* = 0.03. By One-way ANOVA and Tukey correction. KS—Kaposi’s sarcoma, ART—Antiretroviral Therapy, HIV-1—Human Immunodeficiency Virus type 1, NA—Not Applicable, BDL—Below detection limit, *n*—Group sample size, HIV-1-HIV-1 negative, HIV-1^+^ HIV-1 positive.

**Table 2:** Plasma Kaposi’s sarcoma-associated herpes virus (KSHV) and human immunodeficiency virus (HIV)-1 detection by PCR.

ID	KSHV Virion in Plasma (PCR) (Copies/mL)				CD4 Count (Cells/µL)		HIV-1 Plasma Viral Copies/mL	
	Before Treatment	After Treatment	Before Treatment	After Treatment	Before Treatment	After Treatment	Before Treatment	After Treatment
21147	-	-	NA	NA	887	523	NA	NA
21182	+	-	1300	NA	854	1154	NA	NA
21117	+	-	BDL	NA	130	262	3450	BDL
21145	-	-	NA	NA	422	370	BDL	BDL
21161	-	-	NA	NA	710	306	148,000	369,000
21186	+	+	2100	BDL	143	211	34,700	BDL
21220	-	-	NA	NA	167	NR	BDL	BDL
21228	-	-	NA	NA	333	353	BDL	BDL
21119	+	-	BDL	NA	221	244	BDL	BDL
21185	-	-	NA	NA	178	112	BDL	BDL
21120	+	-	BDL	NA	91	482	14,500	BDL
21121	-	-	NA	NA	98	314	6310	BDL
21204	+	-	BDL	NA	150	152	BDL	BDL

+ Positive, − Negative, KSHV—Kaposi’s sarcoma herpes virus, NA—Not applicable, BDL—Below detection limit, HIV-1—Human immunodeficiency virus type 1, Grey shaded—Non-responders, No grey shade —Responders, NR—Not recorded.

## Supplementary Materials

The following are available online at https://www.mdpi.com/2072-6694/12/6/1594/s1. Figure S1: T-cell population analysis from peripheral blood mononuclear cells (PBMCs). Figure S2: T-cell population analysis from peripheral blood mononuclear cells (PBMCs).
