# Supplementary material for: Viral and Immunological Analytes are Poor Predictors of the Clinical Treatment Response in Kaposi’s Sarcoma Patients
Source: Cancers (Basel). 2020 Jun 16;12(6):1594. doi: 10.3390/cancers12061594 (PMC7352224; doi:10.3390/cancers12061594)
Supplement: Supplementary file 1 [file cancers-12-01594-s001.pdf]

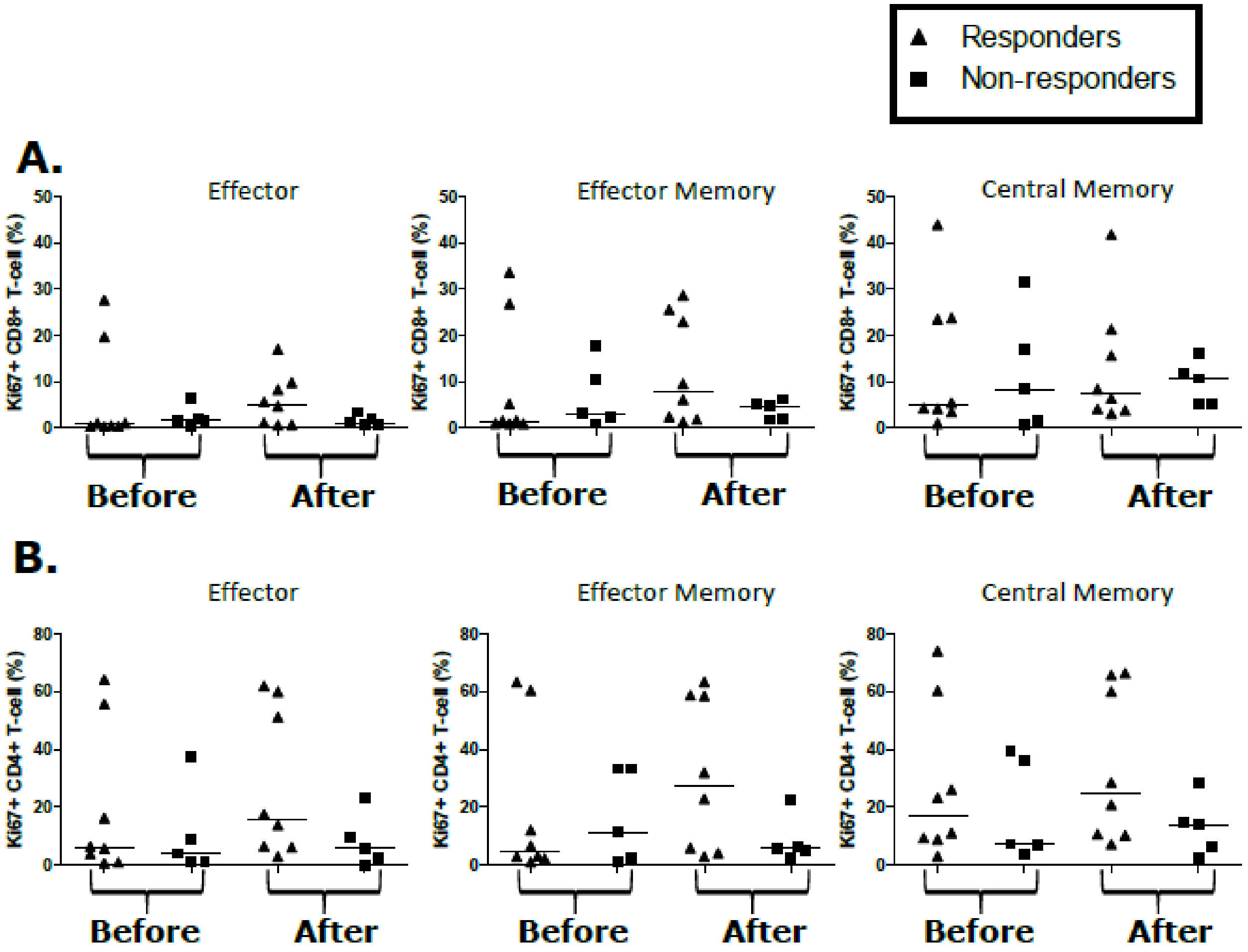

Supplementary Figure S1. T-cell population analysis from peripheral blood mononuclear cells (PBMCs). (**A**) Percentage of $CD8^+$ T-cell expressing proliferation marker ($Ki67^+$) among subsets of $CD8^+$ T-cells in effector, effector memory, and central memory $CD8^+$ T-cells in responders and non-responders before and after KS treatment, and (**B**) percentage of $CD4^+$ T-cell expressing proliferation marker ($Ki67^+$) among subsets of $CD8^+$ T-cells in effector, effector memory, and central memory $CD4^+$ T-cells in responders and non-responders before and after Kaposi's sarcoma (KS) treatment.

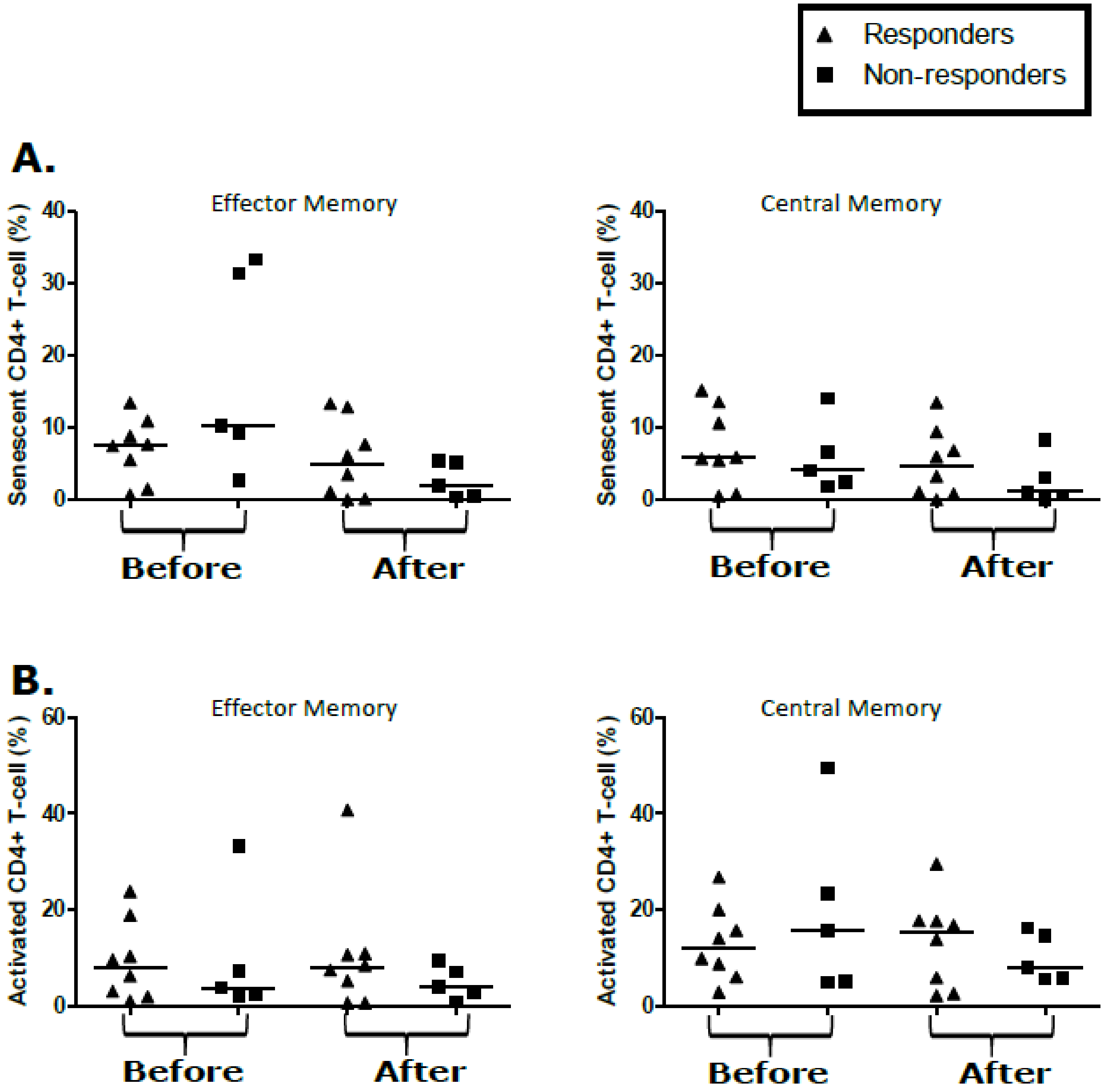

Supplementary Figure S2. T-cell population analysis from peripheral blood mononuclear cells (PBMCs). (**A**) Percentage of $CD4^+$ T-cell expressing senescence markers ($CD57^+$/$hCD28^-$/$CD27^-$) among subsets of $CD4^+$ T-cells in effector memory and central memory $CD4^+$ T-cells in responders and non-responders before and after KS treatment. (**B**) Percentage of $CD4^+$ T-cell expressing activation markers ($CD38^+$ and HLA-DR^+) among subsets of $CD4^+$ T-cells in effector memory and central memory $CD4^+$ T-cells in responders and non-responders before and after KS treatment.
